# The Glucocorticoid Receptor Regulates the *ANGPTL4* Gene in a CTCF-Mediated Chromatin Context in Human Hepatic Cells

**DOI:** 10.1371/journal.pone.0169225

**Published:** 2017-01-05

**Authors:** Masafumi Nakamoto, Ko Ishihara, Takehisa Watanabe, Akiyuki Hirosue, Shinjiro Hino, Masanori Shinohara, Hideki Nakayama, Mitsuyoshi Nakao

**Affiliations:** 1 Department of Medical Cell Biology, Institute of Molecular Embryology and Genetics, Kumamoto University, Kumamoto, Japan; 2 Department of Oral and Maxillofacial Surgery, Faculty of Life Sciences, Kumamoto University, Kumamoto, Japan; 3 Priority Organization for Innovation and Excellence, Kumamoto University, Kumamoto, Japan; 4 Core Research for Evolutionary Science and Technology (CREST), Japan Agency for Medical Research and Development, Tokyo, Japan; Osaka University, JAPAN

## Abstract

Glucocorticoid signaling through the glucocorticoid receptor (GR) plays essential roles in the response to stress and in energy metabolism. This hormonal action is integrated to the transcriptional control of GR-target genes in a cell type-specific and condition-dependent manner. In the present study, we found that the GR regulates the *angiopoietin-like 4* gene (*ANGPTL4*) in a CCCTC-binding factor (CTCF)-mediated chromatin context in the human hepatic HepG2 cells. There are at least four CTCF-enriched sites and two GR-binding sites within the *ANGPTL4* locus. Among them, the major CTCF-enriched site is positioned near the *ANGPTL4* enhancer that binds GR. We showed that CTCF is required for induction and subsequent silencing of *ANGPTL4* expression in response to dexamethasone (Dex) and that transcription is diminished after long-term treatment with Dex. Although the *ANGPTL4* locus maintains a stable higher-order chromatin conformation in the presence and absence of Dex, the Dex-bound GR activated transcription of *ANGPTL4* but not that of the neighboring three genes through interactions among the *ANGPTL4* enhancer, promoter, and CTCF sites. These results reveal that liganded GR spatiotemporally controls *ANGPTL4* transcription in a chromosomal context.

## Introduction

The glucocorticoid receptor (GR) is a member of a family of transcription factors that regulate biological processes, such as basal and stress-associated homeostasis, energy metabolism, and the immune response in a cell-type and condition-dependent manner [1, 2]. In the absence of ligand, GR is present in the cytoplasm in a complex with chaperons such as heat-shock proteins. Upon ligand-induced activation, GR dissociates from the complex and translocates to the nucleus, typically by binding to the glucocorticoid response elements (GREs) to activate or repress transcription of target genes. After the gene control, GR dissociates from its ligand or is degraded [2].

Although evidence indicates that the GR regulates gene expression through binding to promoter regions [3–5], recent genome-wide studies reveal that the GR mainly binds to distal enhancer regions [6] to regulate target-gene activity through long-range interactions between the promoter and enhancer [7–9]. The GR target *Lcn2* encoding the acute-phase protein lipocalin-2 is co-regulated through long-range interactions with *Ciz1* located approximately 30-kb upstream from the *Lcn2* GRE [7]. Further, genomic interaction profiling revealed that numerous GREs interact with the *Lcn2* GRE before GR binding [10]. Moreover, the contact loci were enriched in DNase I-hypersensitive sites, including the consensus motif for the CCCTC-binding factor (CTCF). *FKBP51*, which encodes the GR chaperon FKBP51, is regulated by GR-responsive enhancers far from the transcription start site [9]. *FKBP51* and these enhancers are bordered by CTCF-binding sites, which likely contribute to long-range interactions and loop formation. However, whether CTCF contributes to the regulation of GR target genes remains to be determined.

The *angiopoietin-like 4* gene (*ANGPTL4*) is a primary target of GR and is dominantly expressed in the liver and adipose tissue [11–16]. ANGPTL4 is a secreted protein that inhibits extracellular lipoprotein lipase (LPL), which hydrolyzes triglycerides (TGs) to free fatty acids and glycerol [17–19]. Plasma TG levels are reduced in *ANGPTL4*-null mice and are increased in mice that overexpress ANGPTL4 in the liver [20]. *ANGPTL4*-null mice exhibit reductions in glucocorticoid excess-induced changes associated with hypertriglyceridemia, hepatic steatosis, and visceral obesity [15]. These findings establish the role of ANGPTL4 in GR-mediated lipid metabolism. However, the mechanism of the regulation of *ANGPTL4* by CTCF as well as GR has not been investigated.

Higher-order chromosome conformations, such as chromatin looping, mediate long-range physical interactions between distal regulatory elements and their target genes [21]. The chromatin insulator is a genomic boundary element that controls enhancer activity and the formation of chromatin loops [22]. CTCF is an insulator-binding protein that cooperates with the cohesin complex and other chromatin proteins [23–27]. Genome-wide studies show that CTCF binds several tens of thousands of sites in the mammalian genome. Approximately 50% of the CTCF-binding sites reside within intergenic regions, and the others are present near promoters and within gene bodies [28, 29]. Moreover, CTCF-binding sites are frequently located on the border between transcriptionally active and repressed genes and between different histone modification domains [30]. Although most CTCF-binding sites are conserved among tissues, some of the cell and tissue type-specific sites overlap with transcriptional enhancers, suggesting that CTCF adjusts the interaction between the enhancer element and promoter [29]. For example, CTCF/cohesin-mediated higher-order chromatin mediates basal and inducible gene expression [31–34].

Here, we report that GR regulates *ANGPTL4* in a CTCF-mediated chromatin context in the human hepatic carcinoma cell line HepG2. We identified one GR-binding enhancer site adjacent to a liver-specific CTCF-enriched site. Moreover, in HepG2 cells treated with dexamethasone (Dex), CTCF was required for induction and subsequent silencing of *ANGPTL4*, likely via an enhancer–promoter interaction. Therefore, liganded GR can target *ANGPTL4* but not three neighboring genes. Further, induction of *ANGPTL4* transcription was decreased by long-term glucocorticoid treatment. These results indicate that liganded GR spatiotemporally controls the transcription of *ANGPTL4* depending on the chromosomal context.

## Materials and Methods

### Cell Culture

Human hepatocellular carcinoma HepG2 cells were obtained from the Japanese Collection of Research Bioresources Cell Bank (Osaka, Japan). HepG2 cells were cultured in DMEM (Sigma-Aldrich) supplemented 10% (v/v) fetal bovine serum (FBS). The cells were treated with 100 nM Dex (Sigma-Aldrich) after the cells were cultured for 24 to 48 h in DMEM (Sigma-Aldrich) containing 10% (v/v) dextran-coated charcoal (DCC)-treated FBS.

### Quantitative Real-time PCR (qRT-PCR)

Total RNA was isolated from cultured cells with TRIzol (Invitrogen). For cDNA synthesis, 500 ng of total RNA was reverse-transcribed using a High Capacity cDNA Reverse Transcription Kit (Applied Biosystems), according to the manufacturer’s instructions. We used an ABI Prism 7300 (Applied Biosystems) and SYBR Green to perform qRT-PCR. Each experiment was performed at least three times. Expression levels were normalized to those of *36B4* mRNA encoding ribosomal protein, large, P0. Primer sequences are listed in **S1 Table**.

### Gene Knockdown

GL3 (control) and CTCF-specific siRNAs were used as previously reported [31]. The *RAB11B-AS*-siRNA sequence is as follows: 5´-GACCAAAUAACUAAUGAGA(dT)(dT)-3´and 5´-UCUCAUUAGUUAUUUGGUC(dU)(dG)-3´. Cells were transfected for 48 h with siRNAs in the presence of Lipofectamine RNAiMAX (Invitrogen).

### Chromatin Immunoprecipitation Sequencing (ChIP-Seq)

HepG2 cells (2 × 10^7^) were treated with Dex (100 nM) for 3 h and then cross-linked with 1% formaldehyde at room temperature for 10 min. The cells were lysed with the RIPA buffer supplemented with protease inhibitors. The lysates were sonicated using a Branson Bioruptor to yield 200–500 bp DNA fragments. ChIP was performed using anti-glucocorticoid receptor (GR) antibodies (sc-8992; Santa Cruz Biotechnology, Inc.). DNA fragments were collected using Dynabeads Protein A/G (Life Technologies). Purified DNA (20 ng) was used to construct libraries for sequencing using an Ion Fragment Library Kit (Life Technologies). High-throughput sequencing was performed using Proton semiconductor sequencers (Life Technologies) according to the manufacturer's instructions. Proton sequence data were aligned to the human reference genome hg19. Peaks were detected using the MACS algorithm included with Avadis NGS software (Agilent Technologies). GR binding sites were detected according to a cut-off value of P = 10^−5^ and their enrichment by a factor of 10 vs input. ChIP-seq data for CTCF and histone modifications in HepG2 cells were obtained from the ENCODE/Broad Institute via the UCSC Genome Browser website (http://genome.ucsc.edu/). Gene Expression Omnibus (GEO) accession numbers are as follows: GSM733645, CTCF; GSM733743, H3K27Ac; GSM798321, H3K4me1; and GSM733737, H3K4me3.

### ChIP-qPCR Analysis

HepG2 cells were cross-linked with 1% formaldehyde at room temperature for 10 min. Nuclear lysates were sonicated using the Bioruptor to yield DNA fragments of approximately 200–500 bp. ChIP was performed using the antibodies as follows: anti-CTCF (07–729, Millipore), anti-GR (sc-8992, Santa Cruz Biotechnology, Inc.), anti-H3K27ac (ab4729, Abcam), anti-RNA Pol II serine-5 phosphorylation (provided by Dr. Hiroshi Kimura). Rabbit IgG (sc-2027, Santa Cruz Biotechnology, Inc.) served as a control. Purified DNA was used in quantitative PCR analyses performed using an ABI Prism 7300 System (Applied Biosystems) and SYBR Green. The threshold was set to intersect the linear segment of the PCR amplification curve, and the number of cycles (Ct) required to reach the threshold was counted. We generated a standard curve using the input sample and calculated the enrichment by applying the Ct value of each sample. Primer sequences are listed in **S1 Table**. ChIP analysis of RNA Pol II serine-5 phosphorylation included the addition of a phosphatase inhibitor to the reaction.

### Chromosome Conformation Capture (3C) Assay

Formaldehyde cross-linked chromatin from HepG2 cells was digested with DpnII overnight, followed by ligation catalyzed by T4 DNA ligase at 16°C for 1 h. To prepare standard curves, a bacterial artificial chromosome spanning the *ANGPTL4* locus, RPCI11.C-978J4, was digested with Sau3AI, which is insensitive to Dam methylation, followed by random ligation. After reversing the cross-links, genomic DNA was purified using phenol extraction and ethanol precipitation. We used an ABI Prism 7300 (Applied Biosystems) and a Thunderbird SYBR qPCR Mix (Toyobo) to assess the ligated products. The 3C-qPCR data were normalized to the value of the loading control, using internal primers located within *ANGPTL4*. The relative frequencies of interactions between the reference and its physically adjacent site were normalized to 1. We used the Student *t* test to evaluate the results of more than three independent experiments. Primer sequences are listed in **S1 Table.**

### Statistical Analysis

Differences between two groups were analyzed using the Student *t* test. To compare three groups, we performed ANOVA followed by Tukey-Kramer post hoc test. *P* < 0.05 indicates a statistically significant difference.

### Data Availability

ChIP-seq datasets of GR are deposited in the Gene Expression Omnibus (GEO) database under the accession number GSE85343.

## Results

### Distribution of CTCF and GR-enriched Sites within the *ANGPTL4* Locus

**Fig 1:**
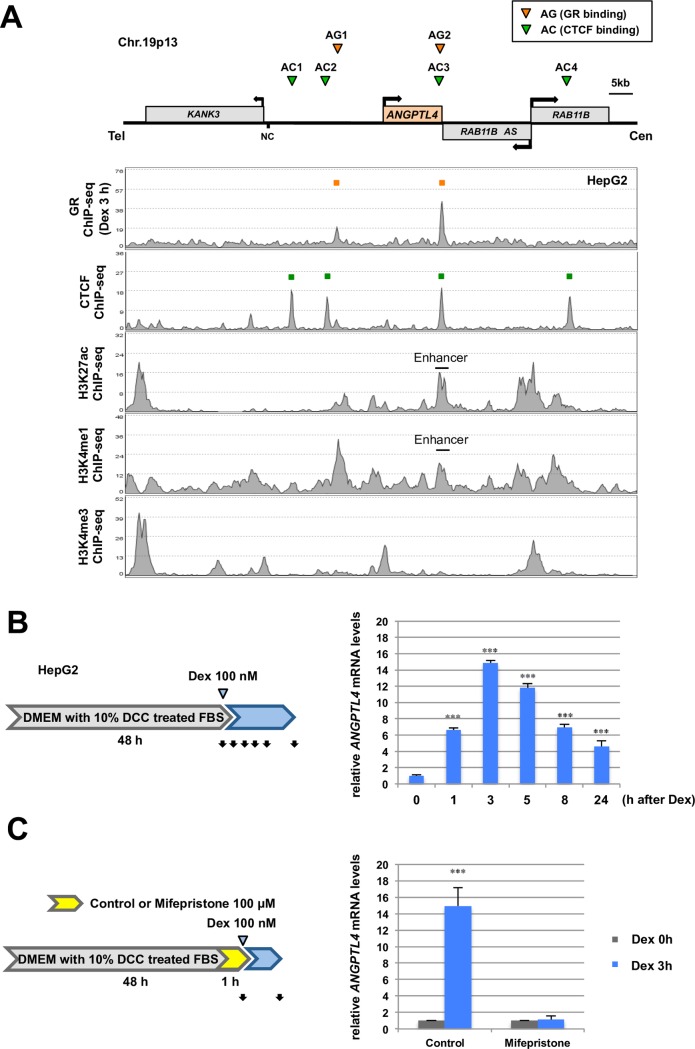
Distribution of glucocorticoid receptor and CTCF in human *ANGPTL4* gene locus. **(A)** Enrichment of the glucocorticoid receptor (GR), CTCF, and modified histone H3 in the *ANGPTL4* locus of HepG2 cells. *KANK3*, *ANGPTL4*, *RAB11B-AS*, and *RAB11B* are located across an approximately 80-kb region. The arrow at the transcription start site of each gene indicates the direction of transcription. According to publically available data and our ChIP-Seq results, two GR-binding sites (designated AG1 and AG2) and four CTCF-enriched sites (designated AC1–AC4) are indicated in orange and green, respectively. NC, negative control. Modifications of histone H3, such as acetylation and methylation, are shown. AG2/AC3 sites are close to each other, and AG2 is an enhancer that has been demonstrated in rat cells [15]. **(B)** Induction of *ANGPTL4* transcription by dexamethasone (Dex). HepG2 cells were grown in DMEM medium supplemented with 10% dextran-coated charcoal (DCC)-treated FBS and were treated with Dex (100 nM). Black arrows show the sampling times of the assays. **(C)** *ANGPTL4* as a direct GR target in HepG2 cells. The GR antagonist mifepristone was added to the medium (100 μM for 1 h) before Dex treatment. The relative expression level is indicated as a value normalized to the level of *36B4* mRNA. Asterisks indicate statistically significance between control (Dex 0 h) and Dex-treated cells at each time point. ***P < 0.005.

The *ANGPTL4* locus is located on human chromosome 19p13 where *KANK3*, *ANGPTL4*, *RAB11B*, and *RAB11B AS* are clustered within an approximately 80-kb region. Among them, *ANGPTL* is a direct target of GR signaling [35]. To examine the chromatin context within this locus, we used publicly available ChIP-Seq data to conduct a survey of genome-wide CTCF-enriched sites in cell lines derived from different tissues. There are at least four CTCF-enriched sites in the *ANGPTL4* locus in HepG2 cells (**Fig 1A**), which contain a CTCF-binding consensus motif (**S1A Fig**). We designated AC1 to AC4. The AC3 site is unique to HepG2 cells (**S2 Fig**), suggesting that AC3 plays a cell-type-specific role in regulating *ANGPTL4* transcription.

We performed ChIP-Seq analysis of HepG2 cells treated with 100 nM Dex for 3 h to identify genome-wide GR binding sites (**Fig 1A**), and identified the GR-enriched sites (AG1 and AG2) in the *ANGPTL4* locus, which possess GREs (**S1A Fig**). ChIP-Seq data indicated that the AG1 and AG2 sites were marked with acetylated lysine residue 27 and mono-methylated lysine residue 4 of histone H3 (H3K27Ac and H3K4me1, respectively), suggesting that they are potential enhancers. These findings are consistent with the evolutionary conservation of the AG2 sequence among rats, mice, and humans, which exhibits GR-responsive enhancer activity in rat H4IIE cells [15]. Further, AG2 and AC3 reside within an approximately 100-bp contiguous region (**S1B Fig**), suggesting that these sites cooperate in the GR-dependent expression of *ANGPTL4*.

### Dexamethasone Induces *ANGPTL4* Expression

We next tested the expression levels of *ANGPTL4* in HepG2 cells treated with Dex. Before Dex addition, the cells were grown in DMEM medium supplemented with 10% DCC-treated FBS for the indicated times to minimize the effects of basal hormone levels. The level of *ANGPTL4* mRNA immediately increased by a factor of 15 at 3 h after Dex addition and decreased from 3 to 24 h (**Fig 1B**). In contrast, Dex treatment did not affect the levels of the three neighboring genes (**S3A Fig**), indicating that *ANGPTL4* is a specific GR target. Western blot analysis showed that ANGPTL4 protein was maximally induced at 3 h after Dex addition and decreased from 3 to 24 h (**S3B Fig**).

To determine whether *ANGPTL4* was directly regulated by liganded GR, we analyzed HepG2 cells treated with the GR antagonist mifepristone (100 μM for 1 h) before Dex treatment. Mifepristone completely inhibited *ANGPTL4* expression 3 h after Dex addition (**Fig 1C**), indicating that *ANGPTL* is the only GR target in this chromosomal region.

### Dexamethasone Treatment Alters Chromatin Status at the *ANGPTL4* Locus

**Fig 2:**
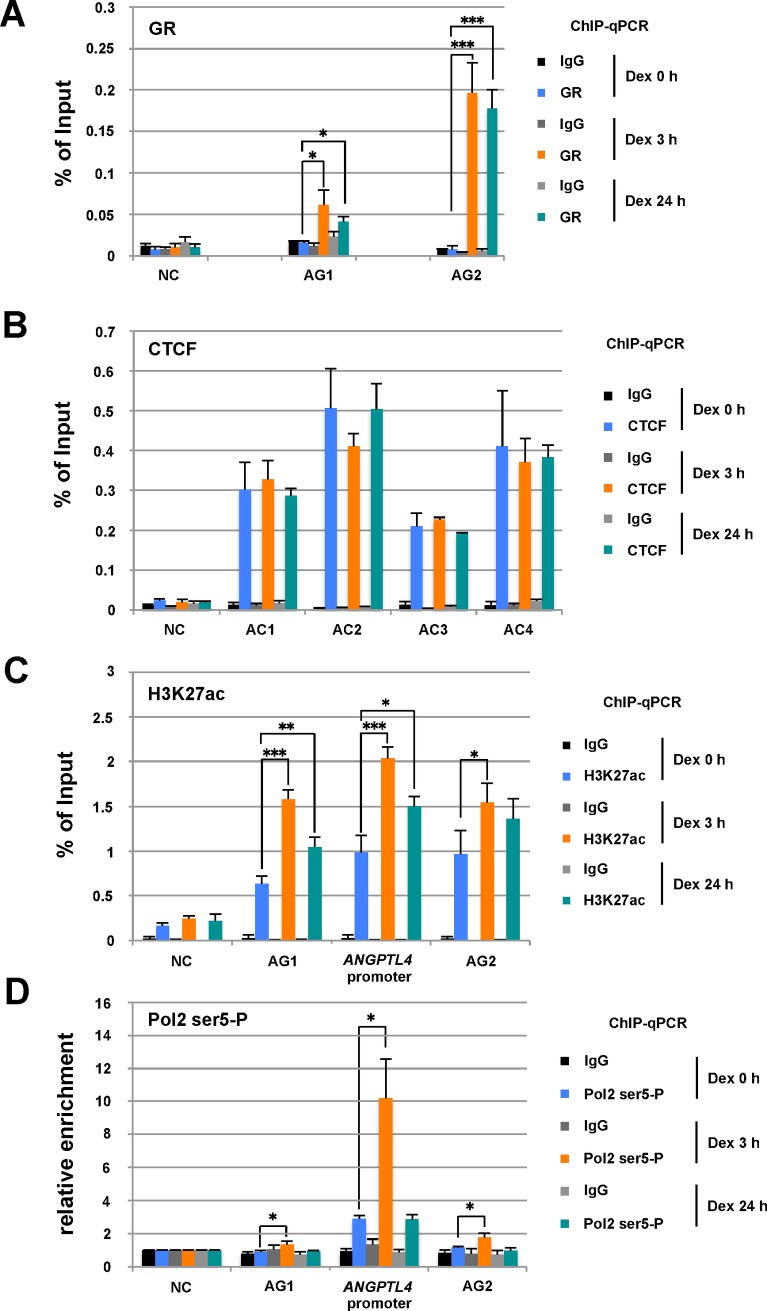
Enrichment of GR, CTCF, acetyl-H3K27, and RNA polymerase II at the *ANGPTL4* gene locus in cells treated with Dex. (A) Enrichment of glucocorticoid receptor (GR) in cells treated with Dex. As shown in **Fig 1B**, HepG2 cells were treated with Dex for 24 h. ChIP-qPCR analysis was performed using an anti-GR antibody and an anti-rabbit IgG (control), followed by quantitative PCR using specific primers for each AG site and the control (NC). (B–D) Enrichment of CTCF, acetyl-H3K27 (H3K27ac), and active RNA polymerase II (Pol2 ser5-P) in cells treated with Dex. ChIP-qPCR analyses were performed using an anti-CTCF antibody and an anti-rabbit IgG (control) (B), anti-H3K27ac (C), and anti-Pol2 ser5-P (D), followed by quantitative PCR using specific primers for each indicated site. Relative enrichment of the control (NC) site was normalized to 1 (D). Asterisks indicate statistically significance between control (Dex 0 h) and Dex-treated cells at each time point. *P < 0.05, **P < 0.01, ***P < 0.005.

We performed ChIP-qPCR analyses to assess the effect of Dex on the chromatin status of the *ANGPTL4* locus (**Fig 2**). We first examined the levels of enrichment of GR at AG1 and AG2. GR was not detected at either AG site under basal conditions (Dex 0 h), while GR bound these AG sites 3 h after Dex addition (Dex 3 h) (**Fig 2A**). GR was similarly retained 24 h after Dex treatment (Dex 24 h), although the levels of *ANGPTL4* mRNA decreased at this time (**Fig 1B**). In contrast, CTCF-enrichment did not significantly change at AC1 to AC4 after Dex treatment (**Fig 2B**), suggesting that GR does not compete with CTCF for DNA binding at the AC3 and AG2 sites, despite their proximity.

We next determined the enrichment of the active chromatin mark H3K27Ac in the *ANGPTL4* promoter and enhancer elements AG1 and AG2 (**Fig 2C**). After Dex treatment for 3 h (Dex 3 h), the H3K27Ac mark increased by a factor of approximately 2 at these sites compared with the basal condition and was detected 24 h after Dex addition. Further, we determined the enrichment of RNA polymerase II (Pol II) at the *ANGPTL4* promoter and enhancers (**Fig 2D**). Active Pol II was enriched at the *ANGPTL4* promoter by approximately a factor of 4 at 3 h after Dex treatment and then decreased to the basal level at 24 h. In contrast, enrichment of Pol II enrichment at AG1 and AG2 was moderately detected 3 h after Dex addition, indicating that liganded GR bound to distal enhancers and activated transcription mediated by the *ANGPTL4* promoter.

### Long-term Dexamethasone Treatment Attenuates *ANGPTL4* Induction and Down-regulates GR Expression

**Fig 3:**
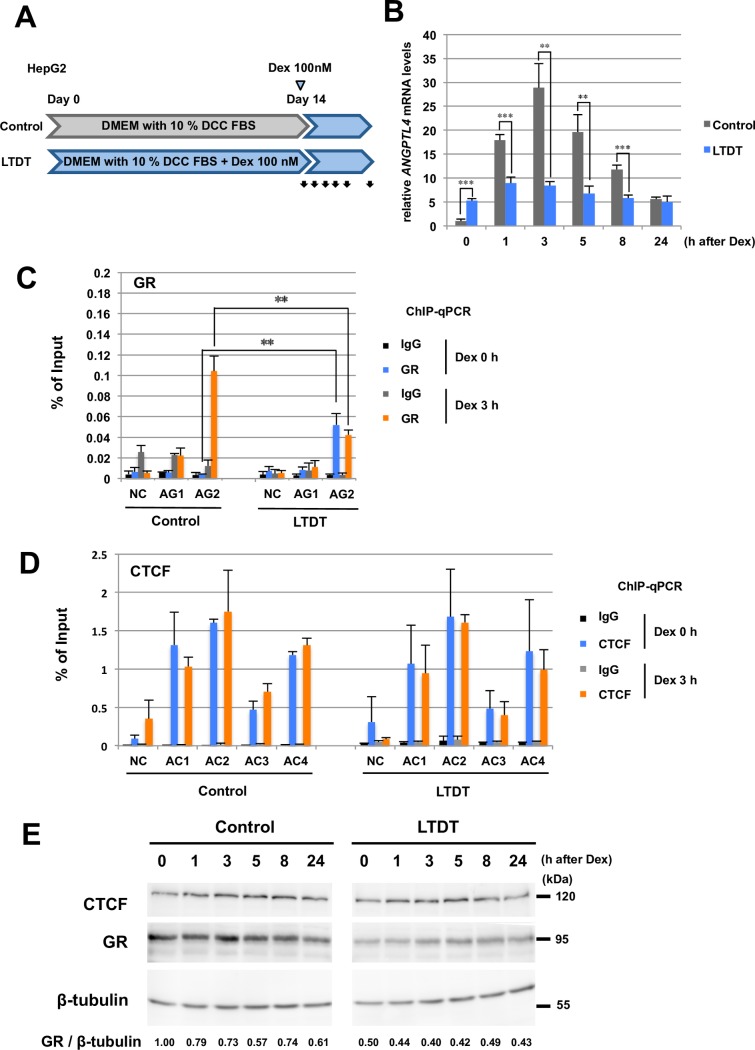
Long-term dexamethasone treatment inhibits the induction of *ANGPTL4* transcription, together with down-regulation of the GR. **(A)** Protocol for Long-Term Dexamethasone Treatment (LTDT). For LTDT, HepG2 cells were initially cultured in DMEM medium supplemented with 10% DCC-treated FBS and 100 nM dexamethasone (Dex) for 14 days. Black arrows show sampling times. (B) Decrease in *ANGPTL4* induction in LTDT cells. **(C)** Decreased enrichment of GR after Dex treatment of LTDT cells. ChIP-qPCR analysis was performed using an anti-GR antibody and an anti-rabbit IgG (control), followed by quantitative PCR using specific primers for each AG site and the control (NC). (D) CTCF enrichment in control and LTDT cells. ChIP-qPCR analysis was performed using an anti-CTCF antibody and anti-rabbit IgG (control), followed by quantitative PCR using specific primers for each AC site. (E) Expression of CTCF and GR after Dex treatment of control and LTDT cells. The amount of GR decreased in most LTDT cells. The relative level of GR normalized to that of β-tubulin is shown below. Uncropped image of western blot analysis is shown in **S6 Fig**. Asterisks indicate statistically significance between control and LTDT cells at each time point. **P < 0.01, ***P < 0.005.

An excess of endogenous or exogenous glucocorticoid causes hypertriglyceridemia, insulin resistance, fatty liver, hepatic steatosis, and obesity as well as Cushing’s syndrome [36]. These changes may be associated, in part, with ANGPTL4 levels. Therefore, we examined whether Long-Term Dex Treatment (shortly LTDT; 100 nM for 14 days) altered *ANGPTL4* expression (**Fig 3A**). Control HepG2 cells were cultured in DMEM medium containing 10% DCC-treated FBS. In control cells, *ANGPTL4* expression increased by approximately 30-fold 3 h after Dex addition and then gradually decreased at 24 h (**Fig 3B**). *ANGPTL4* induction in response to Dex treatment was significantly diminished in the LTDT cells (maximally 1.7-fold at 1 h). The basal level of *ANGPTL4* expression in LTDT cells was higher by a factor of approximately 5, compared with that of the control cells.

We next examined GR enrichment at AG sites in the control and LTDT cells (**Fig 3C**). GR enrichment at AG2 differed significantly between these conditions. GR did not bind AG2 under basal conditions (Dex 0 h) in control cells and was significantly enriched by Dex treatment (Dex 3 h). In contrast, moderate GR binding at AG2 in LTDT cells was detected initially (Dex 0 h), and the levels were approximately 50% of those of the Dex-treated control cells and did not increase after Dex addition (Dex 3 h). Thus, newly added Dex reduced *ANGPTL4* induction in LTDT cells and did not increase GR binding to AG2.

We next checked whether CTCF was involved in the reduction of GR enrichment at AG2 in LTDT cells (**Fig 3D**). ChIP-qPCR analysis using anti-CTCF antibodies revealed that the amounts of CTCF at the AC sites were comparable in the control and LTDT cells. Moreover, we did not detect significant changes in *GRα* (encoding GR) and *CTCF* mRNAs in control and LTDT cells (**S4A Fig**).

To clarify the mechanism of reduced GR binding to AG2 in LTDT cells, we performed western blot analysis of whole-cell lysates and found that the amount of GR significantly decreased in LTDT cells (**Fig 3E**). Further, we detected a decrease in the level of GR in nuclear extracts prepared from LTDT cells (**S4B Fig**), suggesting that a negative feedback mechanism regulates GR levels in LTDT cells. Together, these results suggest that GR targeted *ANGPTL4* and the interaction was affected by long-term exposure of cells to excess concentrations of Dex.

### Loss of CTCF Deregulates *ANGPTL4* Expression Induced by DEX

**Fig 4:**
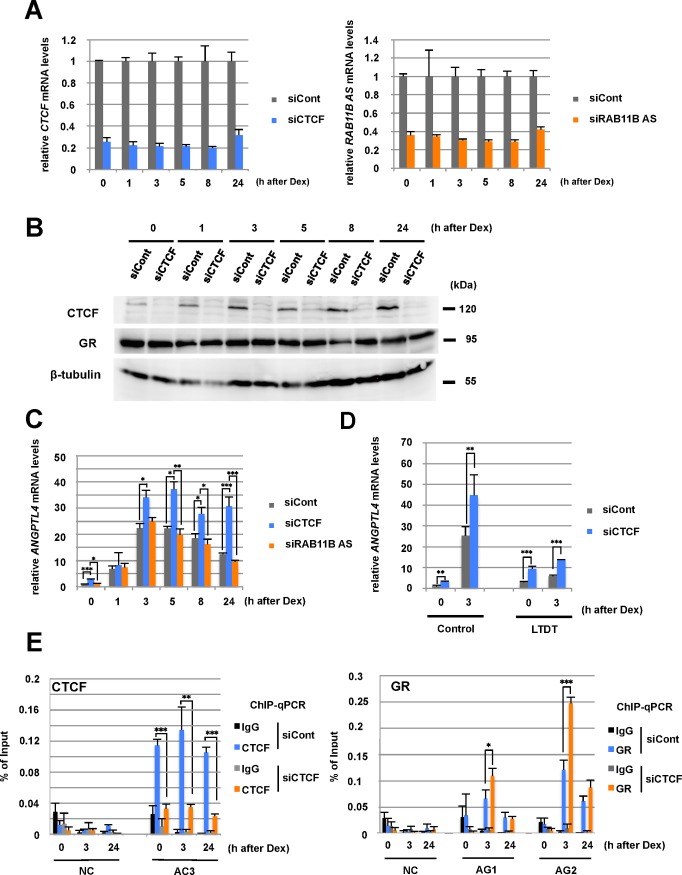
The role of CTCF in regulating *ANGPLT4* transcription. (A) qRT-PCR analysis of HepG2 cells transfected with the siRNAs (siCont, siCTCF, and si*RAB11B AS*) for 48 h and then treated with Dex (100 nM). Expression levels were normalized to those of *36B4* transcripts. **(B)** Western blot analysis of CTCF and GR expression in siRNA-transfected cells. Asterisks indicate statistically significance among siRNA-transfected cells at each time point. **(C)** qRT-PCR analysis of *ANGPTL4* mRNA expression in siRNA-transfected HepG2 cells treated with Dex (see **Fig 4A**). **(D)** qRT-PCR analysis of *ANGPTL4* mRNA expression in siRNA-transfected LTDT cells treated with Dex. Expression levels were normalized to those of *36B4* transcripts. **(E)** Enrichment of CTCF at AC3 and GR at AG sites in siRNA-transfected cells. ChIP-qPCR analysis was performed using anti-CTCF, anti-GR, and anti-rabbit IgG (control) antibodies, followed by quantitative PCR using primers specific for each site. Asterisks indicate statistically significance between control and CTCF-knockdown cells at each time point. *P < 0.05, **P < 0.01, ***P < 0.005.

To determine the role of CTCF in the regulation of *ANGPTL4* transcription, we performed RNA interference-mediated knockdown of *CTCF* expression in HepG2 cells. We conducted qRT-PCR and western blot analyses to confirm the reduction in the levels of *CTCF* mRNA and protein, respectively (**Fig 4A**, left and **4B**). In addition, the depletion of CTCF did not affect GR expression at the protein level (**Fig 4B** and **S5A Fig**). When the CTCF knockdown cells were treated with Dex, *ANGPTL4* mRNA levels significantly increased 3 h after Dex addition, and induction was maintained for 24 h (**Fig 4C**). In contrast, *ANGPTL4* expression increased 3 h after treatment and then decreased in control cells. Similarly, the increased level of *ANGPTL4* mRNA in the CTCF-knockdown cells was detected in LTDT cells (**Fig 4D**). Moreover, we confirmed that the expression of another GR-target gene *FKBP5* was hardly affected by CTCF knockdown (**S5A Fig**).

Several lines of evidence suggest that antisense transcripts influence sense-strand transcription [37]. Further, it has been reported that CTCF has large RNA interactions in mammalian cells [38, 39]. In the *ANGPTL4* locus, the direction of *RAB11B-AS* transcription is opposite of that of *ANGPTL4* and *RAB11B* (**Fig 1A** and **S5B Fig**) and extends to the AC3/AG2 sites in the 3´-region of *ANGPTL4* gene (**S5C Fig**). To test whether the *RAB11B-AS* transcripts influenced *ANGPTL4* expression, we inhibited the expression of *RAB11B-AS* in HepG2 cells (**Fig 4A**, right) and found that Dex-induced *ANGPTL4* expression was unchanged (**Fig 4C**).

We next performed ChIP-qPCR analysis of CTCF-knockdown HepG2 cells to determine whether depletion of CTCF affected GR binding to the AG1 and AG2 sites. (**Fig 4E**). We detected a significant reduction of CTCF enrichment at AC3, and the loss of CTCF caused an increase in GR binding to AG1 and AG2 by a factor of approximately 2 at 3 h after Dex treatment. These data suggest that CTCF negatively regulated the induction of *ANGPTL4* transcription by reducing the amount of liganded GR bound to AG sites. Further, at 24 h after Dex addition, GR enrichment at the AG sites was equivalent in the control and CTCF-knockdown cells, although *ANGPTL4* was expressed at a high level when CTCF was depleted (**Fig 4C**). Moreover, the decrease in the level of *ANGPTL4* mRNA coexisted with a high level of GR binding to the AG sites 24 h after Dex addition (**Figs 1B** and **2A**). Together, these results suggest that Dex-induced *ANGPTL4* expression was regulated by the CTCF-mediated chromatin context as well as by GR binding to the enhancers. We also observed that the expression level of nearby genes of *ANGPTL4* was partly increased by CTCF knockdown (**S5D Fig**), suggesting that the activity of the *ANGPTL4* enhancer may spread to nearby genes in the CTCF-knockdown cells. Thus, CTCF was required for proper induction and subsequent silencing of *ANGPTL4* in cells treated with Dex.

### Dexamethasone-induced Chromatin Conformation Changes in the *ANGPTL4* locus

**Fig 5:**
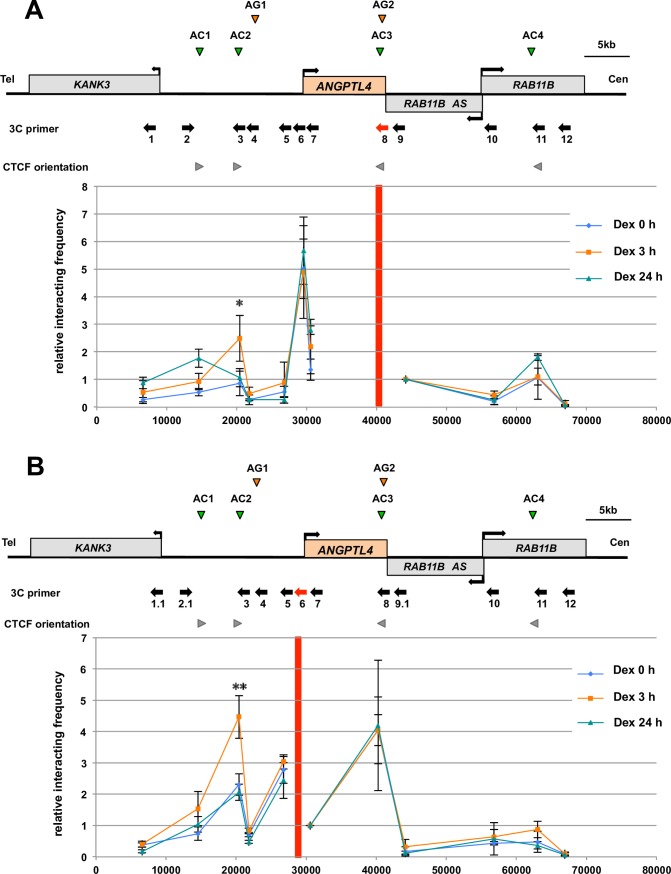
Specific changes in higher-order chromatin conformation of the *ANGPTL4* locus in cells treated with dexamethasone. (A) Chromosome conformation capture (3C) assays were performed using of DpnII-digested fragments containing each AC/AG site and the *ANGPTL4* promoter. AC3/AG2 sites reside in the same fragment, and AG2 is the *ANGPTL4* enhancer [15]. The relative interaction frequencies of the reference AC3/AG2 fragment (indicated with red) with other DpnII fragments were determined using qPCR analysis of at least three distinct samples from HepG2 cells treated with Dex. Gray arrowheads indicate the orientation of CTCF-binding sites. (B) The relative interaction frequencies of the reference *ANGPTL4* promoter (indicated with red) with other DpnII fragments in Dex-treated cells. PCR amplification using internal primers derived from the *ANGPTL4* locus was used as a loading control to normalize the amount of DNA fragments. The efficiencies of DpnII digestions and subsequent ligations were determined at each restriction site. The relative frequencies of interactions between the reference and its closest site in the control state (Dex 0 h) were normalized to 1. Asterisks indicate statistically significance between control (Dex 0 h) and Dex-treated cells (Dex 3h). *P < 0.05, **P < 0.01.

To investigate higher-order chromatin regulation in the *ANGPTL4* locus, we performed the chromosome conformation capture (3C) assay of Dex-treated HepG2 cells. The experiments were performed under the conditions as follows: basal condition (Dex 0 h), Dex treatment for 3 h (Dex 3 h) and 24 h (Dex 24 h). There are at least four CTCF-enriched sites (AC1–AC4) and two GR-binding enhancer sites (AG1 and AG2) in the *ANGPTL4* locus (**Figs 1A** and **5**). DpnII was used to convert these sites and the *ANGPTL4* promoter into distinct fragments, except for the composite AC3/AG2 site. Using quantitative PCR analyses of the intramolecular ligation products, we first tested the interaction frequency of AC3/AG2 as a reference, which is the counterpart of a previously demonstrated enhancer element [15], with the other 11 DpnII fragments. The interaction frequency between AC3/AG2 and the *ANGPTL4* promoter was consistently high under basal and Dex conditions (**Fig 5A**). The interaction between AC3/AG2 and AC2 increased by a factor of 2.5 at 3 h after Dex addition and then decreased to the basal level at 24 h. Dex treatment induced a significant dynamic change at AC2, because the interaction frequencies of AC3/AG2 with the other sites were largely constant.

To further evaluate the higher-order chromatin conformation in this locus, we performed the 3C assay using the *ANGPTL4* promoter as a reference (**Fig 5B**). The interaction frequency of the *ANGPTL4* promoter with AC2 significantly increased 3 h after Dex addition and then decreased to the basal level at 24 h, similar to case of the reference AC3/AG2 (**Fig 5A**). The interaction of the *ANGPTL4* promoter with other fragments was unchanged during Dex treatment, and the *ANGPTL4* promoter possibly interacted with the AC3/AG2 sites. We repeated the 3C assay of CTCF-knockdown and LTDT cells, but the data were not reproducible because of the higher-order chromatin instability of the *ANGPTL4* locus under these conditions (data not shown). Together, our results suggest that 1) the *ANGPTL4* locus maintains the stable higher-order chromatin conformation under basal conditions and Dex treatment and 2) *ANGPTL4* is selectively activated by liganded GR via the associations among the enhancer, promoter, and CTCF sites.

## Discussion

In the present study, we show that GR regulated *ANGPTL4* in a CTCF-mediated chromatin context in the human hepatic carcinoma cell line HepG2. Liganded GR selectively targeted *ANGPTL4* but not the three neighboring genes via interactions among the enhancer, promoter, and CTCF sites. In CTCF-depleted cells, *ANGPTL4* transcription was more inducible by Dex and was persistently up-regulated for 24 h, indicating that CTCF was required for proper induction and subsequent silencing of transcription. Further, long-term Dex treatment subsequently diminished the induction of *ANGPTL4* transcription. Our results therefore identified the mechanism of the regulation of *ANGPTL4* transcription by CTCF as well as GR.

**Fig 6:**
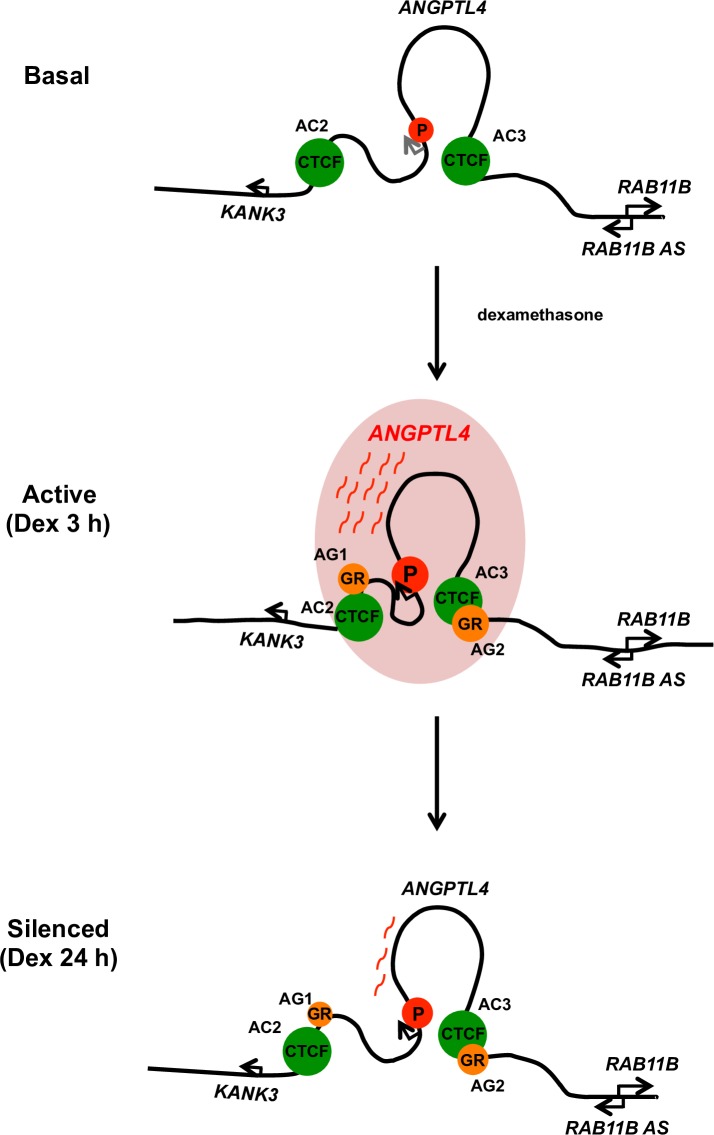
Model of *ANGPTL4* gene regulation and higher-order chromatin in cells treated with Dex. In its basal state, *ANGPTL4* is not activated in the absence of Dex-bound GR within the preexisting higher-order chromatin structure. When *ANGPTL4* is transcriptionally active (Dex 3 h), liganded GR binds to AG sites and enhances interactions between enhancer, promoter, and CTCF sites to selectively induce *ANGPTL4* but not the neighboring *KANK3*, *RAB11B-AS*, and *RAB11B* genes. In the silenced state (Dex 24 h), the positions of enhancer, promoter, and CTCF sites are restored, leading to down-regulation of *ANGPTL4* transcription. CTCF is required for proper induction and subsequent silencing of the *ANGPTL4* transcription.

**Fig 6** shows a model of the regulation of *ANGPTL4* transcription and the conformation of higher-order chromatin in cells treated with Dex. In its basal state, *ANGPTL4* is not activated in the absence of glucocorticoid. However, the higher-order chromatin structure of the locus is prearranged. In the transcriptionally active state (Dex 3 h), liganded GR binds to AG sites and increases the affinities of the interactions among enhancer, promoter, and CTCF sites in *ANGPTL4*, which specifically activate *ANGPTL4* but not the neighboring genes *KANK3*, *RAB11B-AS*, and *RAB11B*. Since AC2 and AC3 show convergent orientations to the CTCF-binding sites, these may facilitate the formation of a chromatin loop [40]. In the silenced state (Dex 24 h), the positions of the enhancer, promoter, and CTCF sites are restored to the basal state when *ANGPTL4* transcription is down-regulated. During these processes, CTCF is required for both induction and subsequent silencing of the *ANGPTL4* gene.

Glucocorticoids are essential regulators of lipid homeostasis and energy metabolism, mainly via GR signaling. Dex treatment of HepG2 cells showed that *ANGPTL4* was induced after 3 h and was silenced after 24 h. During this reaction, *ANGPTL4* locus maintained a higher-order chromatin conformation during basal and induced states, together with specific interactions between the *ANGPTL4* enhancer and promoter that were required for selective transcriptional activity. In contrast, under long-term Dex treatment, *ANGPTL4* induction was significantly inhibited, at least in part, by the decrease in the level of GR. When the levels of glucocorticoids are up-regulated *in vivo* [41] under fasting or starvation, lipolysis in adipose tissue releases TGs and free fatty acids to other tissues, including the liver. Glucocorticoids may induce *ANGPTL4* transcription to promote the catabolism. In contrast, long-term elevation of endogenous or exogenous glucocorticoids causes hypertriglyceridemia, insulin resistance, fatty liver, and obesity [36]. Such a long-term response is likely to produce cellular memory against glucocorticoid exposure. These changes may partly correlate with the level of ANGPTL4, because we show here that this protein was down-regulated in LTDT cells.

The siRNA-mediated inhibition of *CTCF* expression in HepG2 cells shows that *ANGPTL4* transcription was highly induced by 3 h and was subsequently up-regulated for 24 h after Dex addition. Since there was no difference in liganded GR binding 24 h after Dex addition to control and CTCF-depleted cells, enhancer-promoter interactions in *ANGPTL4* may contribute to persistent activation. Moreover, CTCF-binding is known to be affected by DNA methylation in the genome [42, 43], and CTCF protects adjacent sequences against *de novo* CpG methylation[44, 45]. Indeed, *IGF2* imprinting is altered in aged and senescent human epithelial cells [46]. Our previous study shows that CTCF is down-regulated during cellular senescence [33]. Thus, the age-dependent increase of abnormal lipid metabolism may be linked to a reduction in CTCF-mediated regulation of *ANGPTL4* transcription, possibly caused by changes in DNA methylation and the down-regulation of CTCF levels. In conclusion, our study of the human *ANGPTL4* locus sheds light on the molecular basis of the regulation of GR target genes and its biological significance.

## Supporting Information

S1 Fig The sequences of CTCF- and GR-enriched sites in the human *ANGPTL4* locus. (A) The consensus motifs of four CTCF-binding sequences (AC1–AC4) and two GR-binding sequences (AG1 and AG2). **(B)** The close localization of AC3 with AG2 in the 3’-region of *ANGPTL4*.
(TIF)

S2 Fig CTCF-enriched sites in the *ANGPTL4* locus in cultured human cell lines. ChIP-Seq data reveal four major CTCF-enriched sites in the *ANGPTL4* locus. The CTCF-enriched AC3 site is uniquely present in the genome of HepG2 cells.
(TIF)

S3 Fig The expression of four genes within human *ANGPTL4* locus in cells treated with dexamethasone. (A) The expression patterns the *KANK3*, *ANGPTL4*, *RAB11B*, and *RAB11B AS* genes in cluster. In cells treated with Dex, *ANGPTL4* mRNA was only induced by Dex. **P < 0.01. (B) Western blot analysis of ANGPTL4 expression in HepG2 cells under the presence or absence of Dex.
(TIF)

S4 Fig GR and CTCF expression in control cells and cells subjected to long-term dexamethasone treatment (LTDT). (A) Analysis of the expression of glucocorticoid receptor (*GRα*) and *CTCF* mRNAs in LTDT cells. In the presence of Dex, there were no significant differences in the levels *GRα* and *CTCF* transcripts in control and LTDT cells. *P < 0.05. (B) Western blot analysis of GR expression in LTDT cells. Uncropped image of western blot analysis is shown in **S6 Fig**.
(TIF)

S5 Fig Expression analysis of CTCF knockdown cells. **(A)** qRT-PCR analysis of *GRα* (left) and *FKBP5* (right) in siRNA-transfected HepG2 cells treated with Dex. **(B)** The transcribed sequence of *RAB11B* and *RAB11B AS* in the human *ANGPTL4* locus. The arrow indicates the transcriptional start site. **(C)** Close localization of AC3/AG2 with the 3´-region of *RAB11B AS*. **(D)** qRT-PCR analysis of four genes within human *ANGPTL4* locus in siRNA-transfected HepG2 cells treated with Dex. The relative expression level is indicated as a value normalized to the level of *36B4* mRNA. Asterisks indicate statistically significance between control and CTCF-knockdown cells at each time point. *P < 0.05, **P < 0.01, ***P < 0.005.
(TIF)

S6 Fig Uncropped images of western blot analysis. **(A)** Uncropped images of **Fig 3E**. **(B)** Uncropped image of **S4B Fig**.
(TIF)

S1 Table Primers used in this study. (PDF)
