# Supplementary figures and images for: The Glucocorticoid Receptor Regulates the ANGPTL4 Gene in a CTCF-Mediated Chromatin Context in Human Hepatic Cells
Source: PLoS One. 2017 Jan 5;12(1):e0169225. doi: 10.1371/journal.pone.0169225 (PMC5215901; doi:10.1371/journal.pone.0169225)

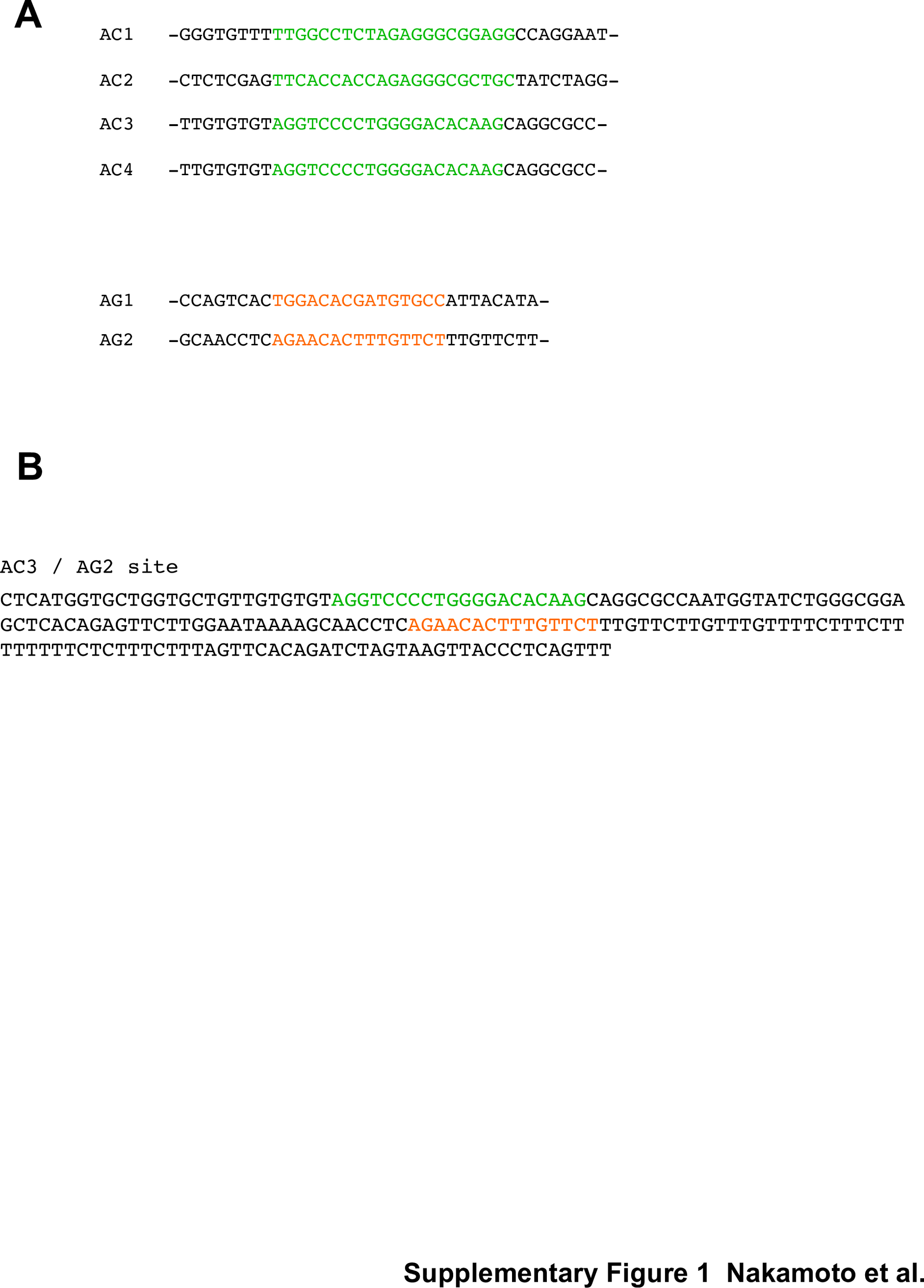

Supplement: S1 Fig — (A) The consensus motifs of four CTCF-binding sequences (AC1–AC4) and two GR-binding sequences (AG1 and AG2). (B) The close localization of AC3 with AG2 in the 3’-region of ANGPTL4. (TIF) [file pone.0169225.s001.tif]

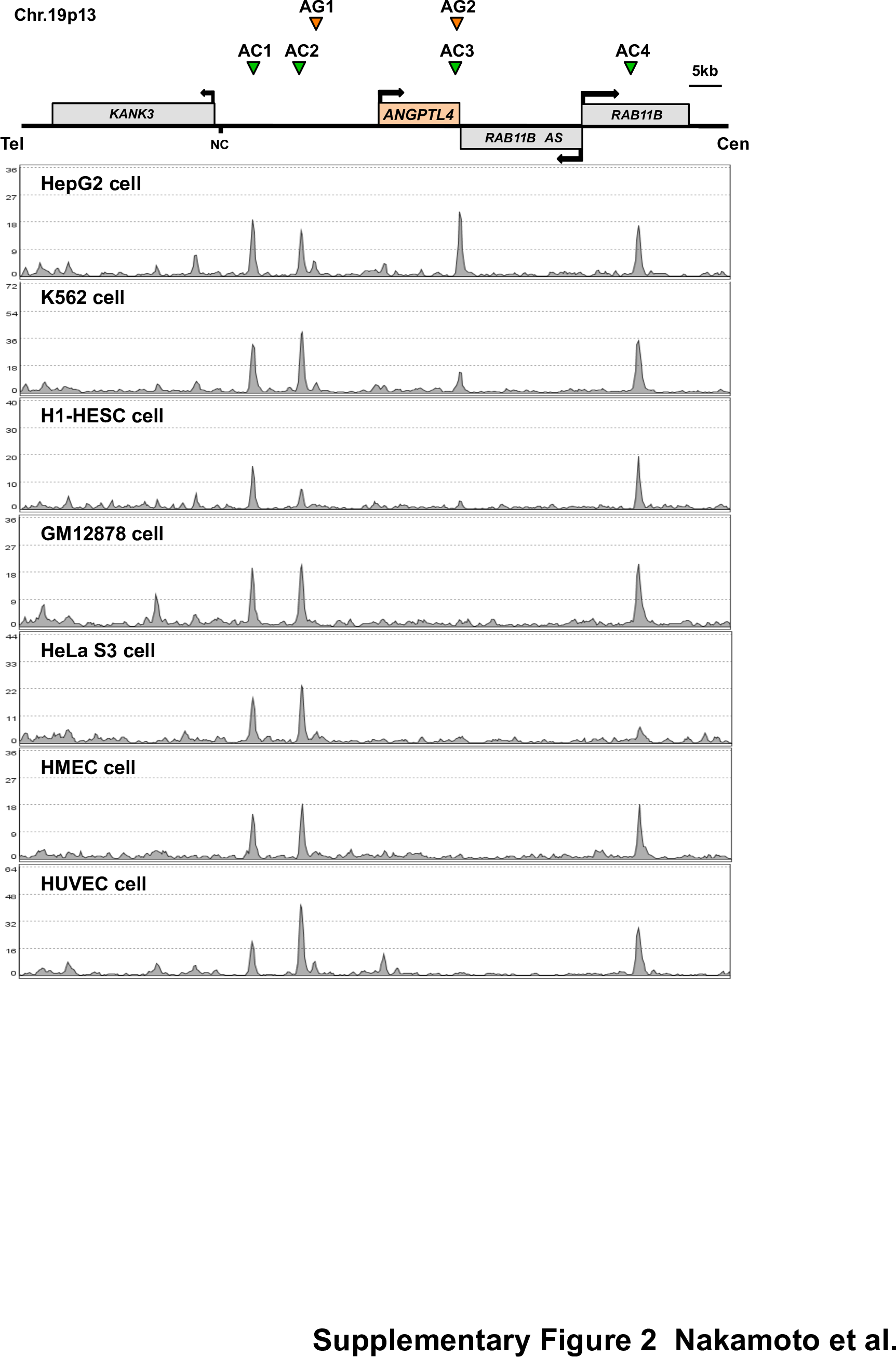

Supplement: S2 Fig — ChIP-Seq data reveal four major CTCF-enriched sites in the ANGPTL4 locus. The CTCF-enriched AC3 site is uniquely present in the genome of HepG2 cells. (TIF) [file pone.0169225.s002.tif]

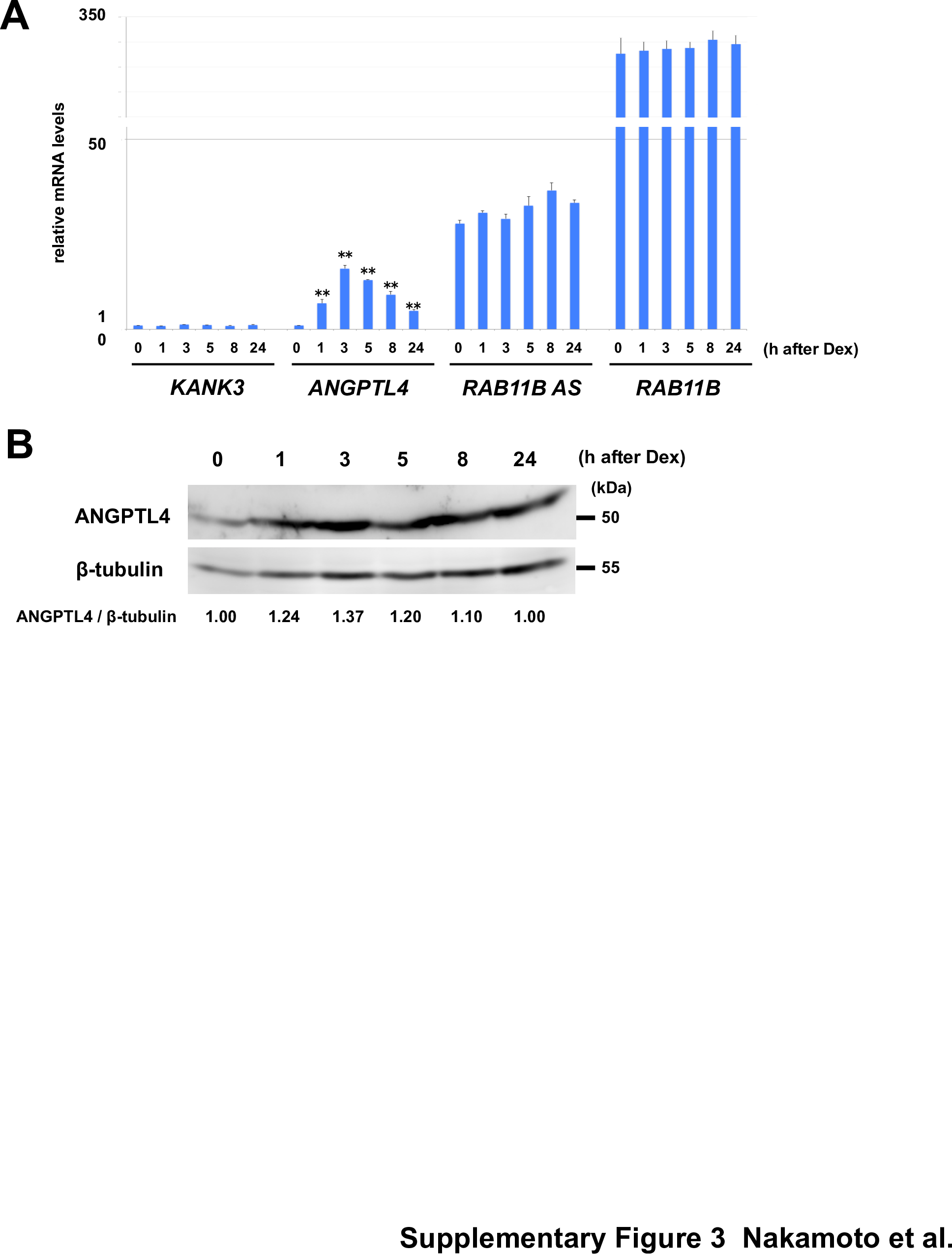

Supplement: S3 Fig — (A) The expression patterns the KANK3, ANGPTL4, RAB11B, and RAB11B AS genes in cluster. In cells treated with Dex, ANGPTL4 mRNA was only induced by Dex. **P < 0.01. (B) Western blot analysis of ANGPTL4 expression in HepG2 cells under the presence or absence of Dex. (TIF) [file pone.0169225.s003.tif]

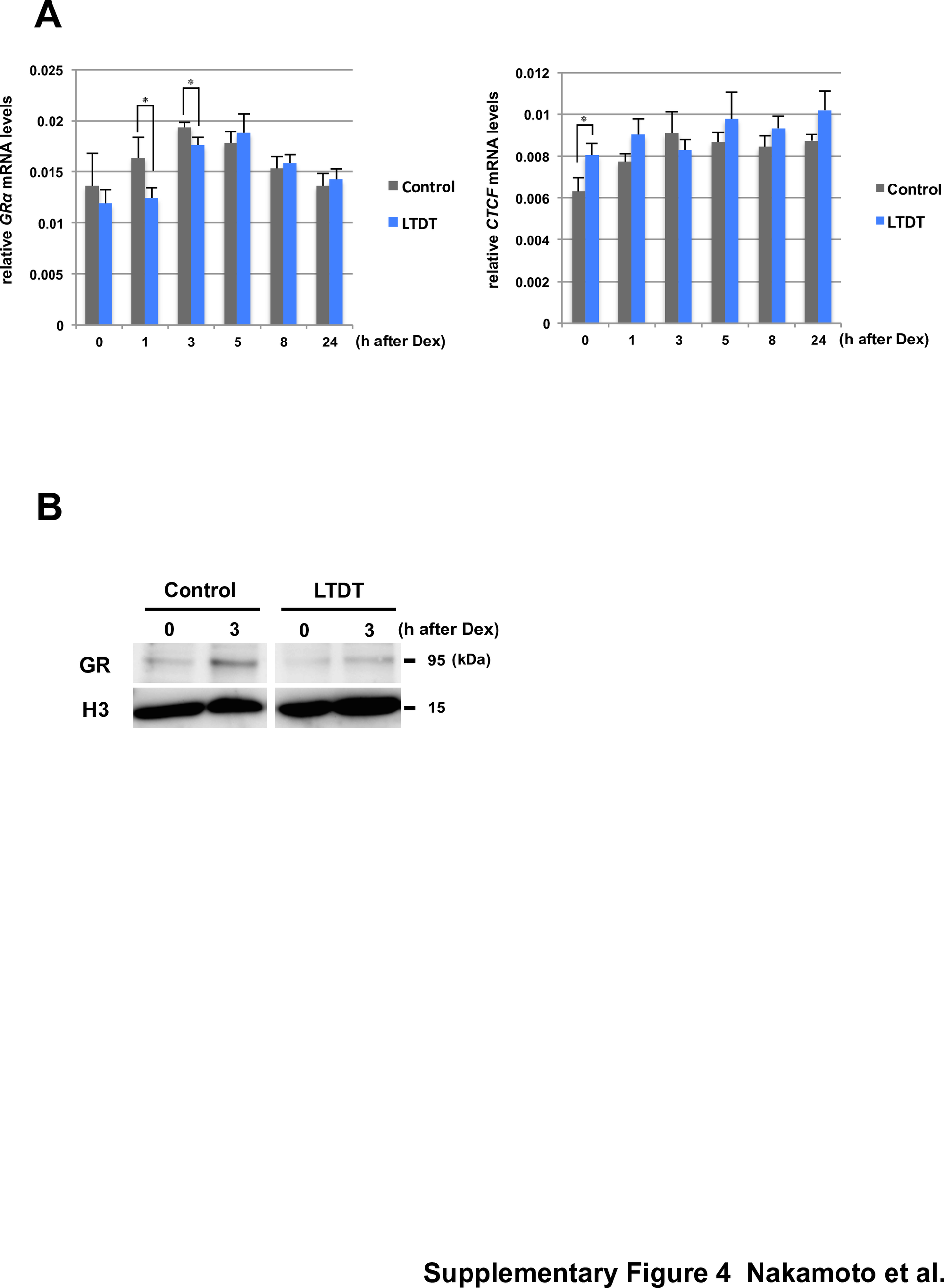

Supplement: S4 Fig — (A) Analysis of the expression of glucocorticoid receptor (GRα) and CTCF mRNAs in LTDT cells. In the presence of Dex, there were no significant differences in the levels GRα and CTCF transcripts in control and LTDT cells. *P < 0.05. (B) Western blot analysis of GR expression in LTDT cells. Uncropped image of western blot analysis is shown in S6 Fig. (TIF) [file pone.0169225.s004.tif]

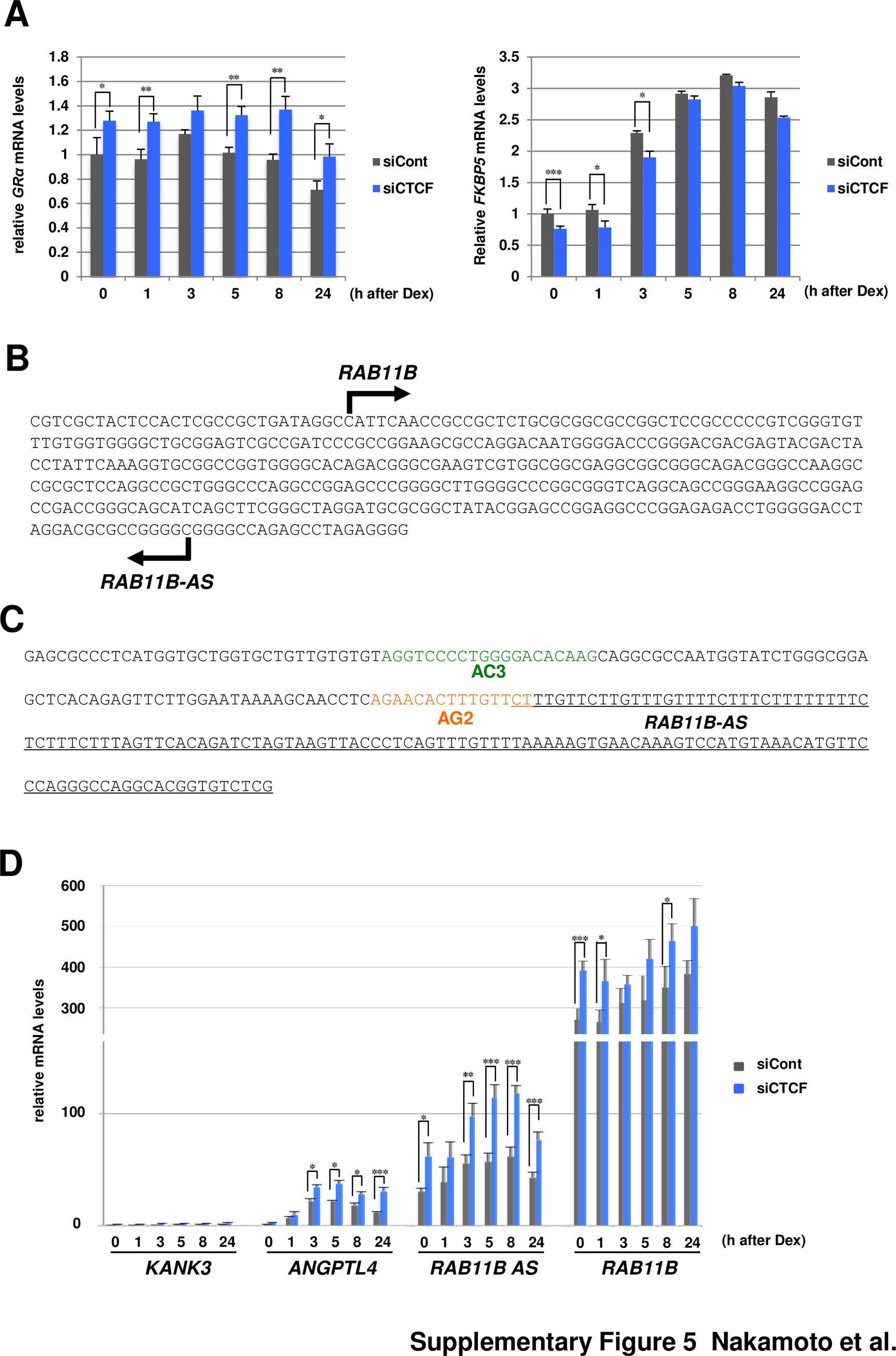

Supplement: S5 Fig — (A) qRT-PCR analysis of GRα (left) and FKBP5 (right) in siRNA-transfected HepG2 cells treated with Dex. (B) The transcribed sequence of RAB11B and RAB11B AS in the human ANGPTL4 locus. The arrow indicates the transcriptional start site. (C) Close localization of AC3/AG2 with the 3´-region of RAB11B AS. (D) qRT-PCR analysis of four genes within human ANGPTL4 locus in siRNA-transfected HepG2 cells treated with Dex. The relative expression level is indicated as a value normalized to the level of 36B4 mRNA. Asterisks indicate statistically significance between control and CTCF-knockdown cells at each time point. *P < 0.05, **P < 0.01, ***P < 0.005. (TIF) [file pone.0169225.s005.tif]

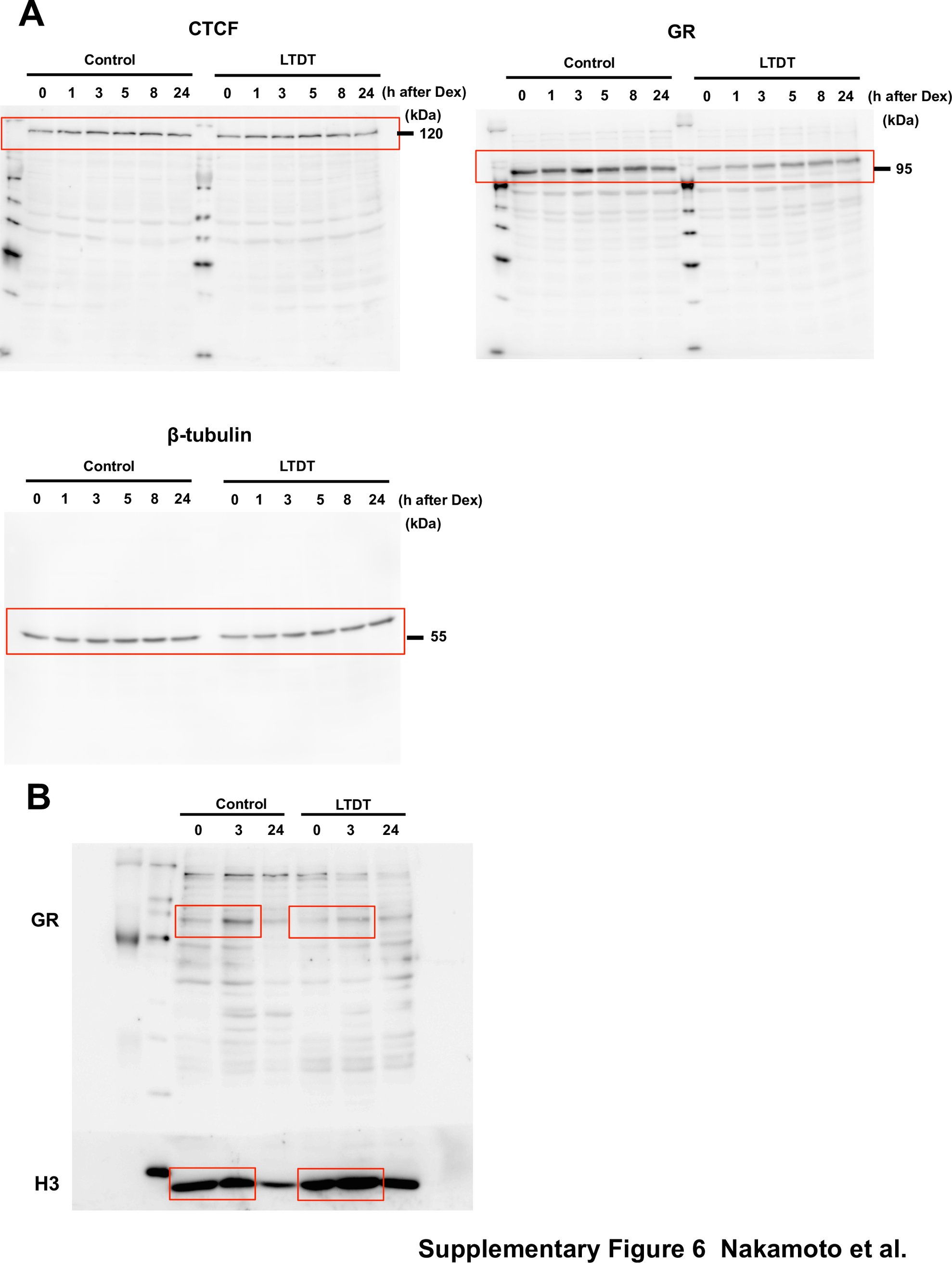

Supplement: S6 Fig — (A) Uncropped images of Fig 3E. (B) Uncropped image of S4B Fig. (TIF) [file pone.0169225.s006.tif]
