# Supplementary material for: The Glucocorticoid Receptor Regulates the ANGPTL4 Gene in a CTCF-Mediated Chromatin Context in Human Hepatic Cells
Source: PLoS One. 2017 Jan 5;12(1):e0169225. doi: 10.1371/journal.pone.0169225 (PMC5215901; doi:10.1371/journal.pone.0169225)
Supplement: S1 Table — (PDF) [file pone.0169225.s007.pdf]

S1 Table. Primers used in this study.

Real time PCR primers

ANGPTL4 Forward	5'-GAGGTCCTTCACAGCCTGCA-3'
ANGPTL4 Reverse	5'-TGGGCCACCTTGTGGAAGAG-3'
KANK3 Forward	5'-CCTGCACTACAGTGTGTCCC-3'
KANK3 Reverse	5'-CTCACAGAGGTGAGTGCAGC-3'
RAB11B Forward	5'-GGACGACGAGTACGACTACC-3'
RAB11B Reverse	5'-TGCTCTCCAGGTTGAACTCG-3'
RAB11B AS Forward	5'-CTTCAAGAATGAAGCCATGG-3'
RAB11B AS Reverse	5'-TGAGGTCAGCAGTTCGAGAC-3'
CTCF Forward	5'-CTTTGCAGCCACGGAGAG-3'
CTCF Reverse	5'-TCTCCTTTCCTTTAATAAAAGTTTCG-3'
GRα Forward	5'-AAGGAAACTCCAGCCAGAAC-3'
GRα Reverse	5'-CGGGGAATTCAATACTCATGG-3'
FKBP5 Forward	5'-GAGCAGGGAGAGGATATTACC-3'
FKBP5 Reverse	5'-CTTTGTCTCCAATCATCGGC-3'
36B4 Forward	5'-GATGCCCAGGGAAGACAG-3'
36B4 Reverse	5'-TCTGCTCCCACAATGAAACAT-3'

ChIP-qPCR primers

AC1 Forward	5'-TTGGGTAATGAGGAGGCGGGT-3'
AC1 Reverse	5'-TCTTGGTCCTTGGGGATGGC-3'
AC2 Forward	5'-TCTGAGCGGCCAGAACAGCT-3'
AC2 Reverse	5'-TGTATATACCCGGTTTGACAACCGC-3'
AC3 Forward	5'-TGGGTCGAGAGCGCCCTCAT-3'
AC3 Reverse	5'-AGAACTCTGTGAGCTCCGCCC-3'
AC4 Forward	5'-GGTTGTCTGCAGAGGAGGGA-3'
AC4 Reverse	5'-CGCTCATCACCAACCAAAACGC-3'
AG1 Forward	5'-AGACTGTGGTTAGTTGCTGG-3'
AG1 Reverse	5'-GGATTCTTCTCAGACAAGCCC-3'
AG2 Forward	5'-CTTGGAATAAAAGCAACCTC-3'
AG2 Reverse	5'-GGGTAACTTACTAGATCTGTG-3'
ANGPTL4 promoter Forward	5'-CAAGACTCCTCCGCCCACTC-3'
ANGPTL4 promoter Reverse	5'-CGGATCACAGTCGTGTGAGGA-3'
Negative control Forward	5'-AGAGGTCAAGTCTCTGCCCAG-3'
Negative control Reverse	5'-AGAAATCCCACCGGGTTACTC-3'

<u>3C primers</u>

1	5'-AGACGGAGTCTGGCTCTGTCC-3'
1.1	5'-AGGTCCCTGACCTCTCCCTTC-3'
2	5'-CCCAGGCTGGAGTGCAGTGGC-3'
2.1	5'-TTGCTCAGGCTGGAGTGCGAT-3'
3	5'-TCTGGTGGTGAACTCGAGAGCAGC-3'
4	5'-GTCACACAGCTCAGAAGGGTC-3'
5	5'-GAATGTGGCCTCTTACCCAGC-3'
6	5'-AATTATGAGTGCCTTAGCGGC-3'
7	5'-GTAAGACCCGCTTGGTTGCAG-3'
8	5'-GCAAGGAGTGAAGCTCCATGC-3'
9	5'-CAGTGACAGTGGGCTCAGAGG-3'
9.1	5'-TTCCCAGGGTGTTCATTGGGC-3'
10	5'-AGGTAGTCGTACTCGTCGTCC-3'
11	5'-TTCCCCCAACACGCCAGGTGC-3'
12	5'-GAGAGACAGATGTACCTGAGG-3'
